# Thrombocytosis and bleeding in myeloproliferative neoplasms: exploring clinical diversity and risk of acquired von Willebrand syndrome—insights from a UK center

**DOI:** 10.1016/j.rpth.2025.102954

**Published:** 2025-06-24

**Authors:** Giulia Simini, Andrew Innes, Saravanan Vinayagam, Golzar Mobayen, Nilanthi Karawitage, Simone Claudiani, Zain Odho, Mike Laffan, Deepa J. Arachchillage

**Affiliations:** 1 Department of Haematology, Imperial College Healthcare National Health Service Trust, London, UK; 2 Department of Haematology, Cancer Institute, University College London, London, UK; 3 Centre for Haematology, Department of Immunology and Inflammation, Imperial College London, London, UK; 4 North West London Pathology, Imperial College Healthcare National Health Service Trust, London, UK

**Keywords:** antiplatelet treatment, bleeding, myeloproliferative neoplasms, thrombocytosis, von Willebrand factor

## Abstract

**Background:**

Myeloproliferative neoplasms (MPNs) represent a group of blood disorders characterized by myeloid cell proliferation and an associated increased risk of thrombosis and bleeding. Platelet count may have a direct link to these complications.

**Objectives:**

To share our MPN clinic’s experience with hemostatic testing and bleeding outcomes in patients with platelets ≥ 800 × 10^9^/L.

**Methods:**

This was a single-center retrospective study from May 2022 to September 2024. Clinical characteristics, treatments, and bleeding events of patients with MPN or chronic myeloid leukemia were recorded. Laboratory assessments included full blood count, renal function, coagulation profiles, platelet function test, and von Willebrand factor (VWF) assays.

**Results:**

A total of 39 patients were included, majority of whom received aspirin for thrombosis prevention (76%). The study found that bleeding complications occurred in 33% of patients, with mucocutaneous bleeding being the most common. There was a trend toward bleeding in patients on aspirin (*P* = .07). However, platelet count alone did not predict bleeding risk. While some patients showed abnormal VWF function, low VWF levels were not consistently associated with increased bleeding. Interestingly, we found moderate negative correlation between baseline VWF ristocetin/antigen and activated partial thromboplastin time (*P* = .02; *r* = −.37) and prothrombin time (*P* = .009, *r* = −.45), suggesting other potential coagulation imbalances associated with bleeding diathesis in MPNs. Post cytoreduction, there was a significant increase in mean VWF ristocetin/antigen ratio (*P* = .0009).

**Conclusion:**

The study illustrates the limitations of relying solely on platelet counts to estimate bleeding risk in MPN patients. Assessment of VWF activity and careful selection of antithrombotic therapy were highlighted as important considerations.

## Essentials

• MPNs are associated with an increased risk of thrombosis and bleeding.
• Clinical characteristics, treatments, and bleeding events were assessed in patients with MPN.
• Bleeding complications occurred in 33% of patients, and mucocutaneous bleeding was most common.
• Platelet count and VWF activity were not reliable predictors of bleeding risk in MPN patients.

## 1 Introduction

Myeloproliferative neoplasms (MPNs) are characterized by abnormal proliferation of myeloid blood cells, which carries significant risks of thrombosis and bleeding. Advances in classification using molecular, clinical, and morphological criteria have improved understanding of these disorders and underscored the need for nuanced approaches to risk assessment and management [1]. The management of thrombotic and hemorrhagic risks is critical in treating MPN.

A recent meta-analysis involving 13,436 patients with Philadelphia chromosome-negative MPNs found a 20% overall prevalence of thrombotic events at diagnosis, comprising 16.2% with arterial and 6.2% with venous events. The distribution of these events was 28.6% in polycythemia vera (PV), 20.7% in essential thrombocythemia (ET), and 9.5% in prefibrotic myelofibrosis (PFMF). Additionally, the meta-analysis reported a 6.2% overall prevalence of hemorrhagic events, with similar rates across the subtypes: 8.9% in PFMF, 7.3% in ET, and 6.9% in PV [2].

Despite advances in stratification scores designed to identify patients at higher risk of thrombosis [[3], [4], [5]], there remains considerable variability in clinical practice when it comes to managing their bleeding risk [6,7]. One important risk is the potential for acquired von Willebrand syndrome (AVWS), which can complicate the management of MPN. This is attributed to loss of high-molecular-weight multimers due to increased platelet binding and enhanced *ADAMTS-13* cleavage [8].

Determining the true prevalence of AVWS is challenging due to frequent underdiagnosis, misdiagnosis, and the lack of appropriate laboratory testing required for its detection. A retrospective analysis of 170 ET patients from Mital et al. [9] reported that up to 1 in 5 patients can develop AVWS. The same group also reported AVWS in up to 1 in 10 patients with polycythemia rubra vera in a subsequent retrospective analysis of 142 cases [10].

Another important risk is the use of antiplatelet therapy, which should be considered carefully when platelet count exceeds 1000 × 10^9^/L, alongside other clinical and laboratory factors, due to the clinical heterogeneity among patients [11,12]. A platelet count > 1500 × 10^9^/L can also trigger consideration of cytoreductive treatment to prevent thrombo-hemorrhagic complications [[13], [14], [15]]; however, this notion has been challenged, especially when managing young/low-risk patients [16,17]. Moreover, there is evidence of bleeding complications arising despite a normal or near-normal platelet count [[18], [19], [20]].

This retrospective single-center cohort study aimed to share our MPN clinic’s experience, with a particular emphasis on hemostatic testing and bleeding outcomes in patients with platelet counts ≥ 800 × 10^9^/L.

## 2 Methods

Patients with a diagnosis of MPN or chronic myeloid leukemia, according to World Health Organization criteria [1], and platelet counts ≥ 800 × 10^9^/L were included under an approved local protocol (audit number HAE_021) at Hammersmith Hospital, London, UK, from May 2022 to September 2024. This platelet threshold was agreed upon to capture patients with less extreme thrombocytosis, as well as those with platelet counts > 1000 × 10^9^/L. The proposed mechanisms of AVWS in MPN involve loss of high-molecular-weight multimers [8]. We, therefore, determined von Willebrand factor (VWF) ristocetin cofactor (VWF:RCo)/antigen (Ag) and VWF collagen binding (VWF:CB)/Ag ratios to help detect this phenomenon [21,22]. A reduction of these ratios <0.7 has been regarded as abnormal in previous studies of AVWS by analogy with congenital von Willebrand disease (VWD) [23,24], and <0.6 based on results of healthy controls [9,[25], [26], [27]]. Definition of bleeding events followed the International Society on Thrombosis and Haemostasis major bleeding and clinically significant nonmajor bleeding criteria [28,29]. Data collection included demographics, baseline and posttreatment clinical characteristics, confirmed bone marrow histological diagnosis, and driver mutation analysis. Information on anticoagulation and antiplatelet treatments, relevant medical history (particularly cardiovascular/thrombosis and bleeding risks), and bleeding events, including type, was also gathered. Baseline and posttreatment laboratory assessments included complete blood count, renal function (estimated glomerular filtration rate [eGFR]), activated partial thromboplastin time (aPTT), prothrombin time (PT), fibrinogen, factor (F)VIII activity, VWF antigen (VWF:Ag), VWF:RCo, VWF:CB, VWF multimer analysis, platelet function analysis, and blood group testing. The STA-VWF:RCo Kit (Stago) measured VWF activity by tracking turbidity changes when ristocetin was added to a platelet suspension in the test plasma, with absorbance levels reflecting VWF activity. Similarly, VWF was assessed with the STA-VWF:Ag Kit, which uses turbidity changes in a suspension of latex microparticles coated with VWF antibodies. VWF:CB assay was measured with TECHNOZYM vWF:CBA ELISA Kit (Technoclone) following manufacturer’s protocol.

The k-nearest neighbors imputation strategy was used to account for missing laboratory values (VWF:RCo = 2, VWF:Ag = 2, VWF:CB = 10, and FVIII:C = 9) but not for comorbidities or clinical outcomes. The Fisher’s exact test and Mann–Whitney *U*-test were employed to compare clinical/laboratory variables between the bleeding and nonbleeding groups in univariate analysis. Spearman correlation was used to explore relationship between different variables (GraphPad Prism v10.1.0). Logistic regression was performed in R v4.3.2 (open source software) using the *glm* function. The odds ratios and significance of covariates were determined against bleeding as outcome. The covariates used were as follows: biological sex, age >50 years, primary diagnosis, driver mutation, antiplatelets, anticoagulation, type of treatment, relevant medical history, and baseline blood test results. Statistical significance was defined as *P* < .05 with *P* values adjusted by Bonferroni correction.

## 3 Results and Discussion

A total of 39 patients (64% female) were included, with a median age of 44 years (range, 17-88). Among these patients, 26 had ET, 7 had PV, 3 had myelofibrosis, 2 had *BCR-ABL*–positive chronic myeloid leukemia, and one had an unclassified MPN. The *JAK2 V617F* mutation was present in 22 of 39 patients (56%), while the *CALR* mutation was the second most common, found in 11 patients (28%). Only 1 patient had an *MPL* mutation, while 3 had no identifiable driver mutations.

In terms of blood count control, 5 required venesections alone, 25 received cytoreductive therapy (12 hydroxycarbamide, 6 pegylated interferon, 1 busulphan, and 6 multiple lines of treatment), and 9 had watchful waiting.

With regard to antithrombotic management, antiplatelet monotherapy (aspirin in all cases) was administered to 77% (30/39) of patients. Two patients in this group received intermittent prophylactic anticoagulation during pregnancy alongside aspirin. Two patients were on dual aspirin and anticoagulation therapy: one was on apixaban due to a history of pulmonary embolism, atrial fibrillation, and stroke, and the other was on warfarin for arterial thrombosis and concomitant diagnosis of antiphospholipid syndrome. Additionally, 1 patient was treated with single-agent apixaban for atrial fibrillation, and 6 patients received no antithrombotic therapy.

Approximately one-third of patients had preexisting cardiovascular/thrombotic risk factors, while none had documented preexisting bleeding risk (Table). One patient was later diagnosed with mild Factor V (FV) deficiency and dysfibrinogenemia; however, the relationship of these to MPN remains unclear.

**Table:** Baseline characteristics of study cohort.

Baseline characheristics *(N = 39)*			
Clinical characteristics		Laboratory characteristics	
Biological sex	No of patients	Full blood count	Median (range)
Male	14	Platelets N × 10^9^/L	1168 (800-2435)
Female	25	Hemoglobin g/L	138 (65-170)
		White blood cell count N × 10^9^/L	10.30 (4.52-251)
Age (y)	Median (range)	eGFR mL/min/1.73m^2^	No of patients
	44 (17-88)	>90	25
		60-89	12
		30-59	2
Primary diagnosis	No of patients	Clotting profile	Median (range)
ET	26	APTT sec	33.20 (26.1-48.8)
PRV	7	PT sec	14.40 (11-16.30)
MF	3	Fibrinogen g/L	3.17 (1.84-5.46)
CML	2	FVIII levels IU/mL	0.92 (0.27-1.86)
MPN U	1		
Commorbidities	No of patients	VWF profile	Median (range)
Cardiovascular/thrombotic risk	13	VWF Ag IU/mL	1.03 (0.38-2.11)
Bleeding risk	0	VWF:RCo IU/mL	0.48 (0.15-1.8)
None	26	VWF:CB IU/mL	0.6 (0.15-1.6)
Driver mutation	No of patients	Relevant ratios	Median (range)
JAK2	22	VWF:RCo/Ag	0.55 (0.15-1.07)
CALR	11	VWF:RCo/CB	0.68 (0.15-1.58)
BCR ABL1	2		
MPL	1		
No driver mutation identified	3		
Treatment			
	No of patients	Antithrombotic management	No of patients
Cytoreduction	25	Anticoagulation monotherapy	1
Hydroxycarbamide	12	Antiplatelet monotherapy	30
Peg-interferon	6	Antiplatelets and anticoagulation	2
Busulphan	1	None	6
Multiple lines	6		
Venesection	5		
Monitoring	9		

APTT, activated partial thromboplastin time; CML, chronic myeloid leukemia; ET, essential thrombocythemia; FVIII, factor VIII; eGFR, estimated glomerular filtration rate; MF, myelofibrosis; MPN U, unclassified myeloproliferative neoplasm; PT, prothrombin time; PRV, polycythemia rubra vera; VWF:Ag, von Willebrand Factor antigen; VWF:RCo, von Willebrand factor ristocetin assay; VWF:CB, von Willebrand factor collagen binding assay.

The median platelet count was 1168 × 10^9^/L (range, 800-2435; IQR, 984-1377), the median hemoglobin was 138 g/L (range, 65-170; IQR, 126-149), and the median white blood cell count was 10.30 × 10^9^/L (range, 4.52-251; IQR, 8-13.40). Most patients had either normal renal function (eGFR > 90 mL/min; *n* = 25) or mild renal impairment (eGFR 60-89 mL/min; *n* = 12), while 2 patients had an eGFR < 60 mL/min. Coagulation profiles revealed a median aPTT of 33.20 seconds (range, 26.1-48.8; IQR, 31.30-35.40; reference range, 23.9-35.5 seconds) and a median PT of 14.40 seconds (range, 11-16.30; IQR, 13.50-15.20; reference range, 12.8-17.4 seconds). The median fibrinogen level was 3.17 g/L (range, 1.84-5.46; IQR, 2.54-3.57). VWF multimer analysis showed a normal pattern in all 18 patients tested (Supplementary Figure).

VWF testing revealed a median VWF:Ag of 1.03 IU/mL (range, 0.38-2.11; IQR, 0.76-1.19; reference range, 0.45-1.80 IU/mL). Two patients had VWF:Ag < 0.5 IU/mL, but none had <0.3 IU/mL. The median VWF:RCo was 0.48 IU/mL (range, 0.15-1.80; IQR, 0.38-0.65; reference range, 0.45-1.80 IU/mL), and 20 patients had VWF:RCo < 0.5 IU/mL, of which 5 had VWF:RCo <0.3. The median VWF:CB was 0.60 IU/mL (range, 0.15-1.60; IQR, 0.46-0.76; reference range, 0.45-1.50 IU/mL). The median FVIII activity was 0.92 IU/mL (range, 0.27-1.86; IQR, 0.71-1.17). A total of 32 (82%) patients presented a VWF:RCo/Ag ratio <0.7 at baseline. Only a minority of patients (10/39; 25%) had their blood group tested, and 7 of them were group O. None of these group O patients had a reduced VWF:Ag.

Among the 14 patients who underwent platelet function testing, 5 were still on aspirin at the time of the test. Of the remaining 9, 4 showed flow obstruction due to elevated platelet counts, 2 had prolonged collagen/adenosine diphosphate closure times, 1 had prolonged collagen/epinephrine closure times, and one had both abnormalities. Only one patient had a normal result.

Thirteen patients (33%) experienced at least one bleeding episode, which included mucocutaneous bleeding (*n* = 7), gastrointestinal bleeding (*n* = 2), heavy menstrual bleeding (*n* = 2), postexertional muscle hematoma (*n* = 1), subconjunctival hemorrhage (*n* = 1), and hemarthrosis (*n* = 1). Of these, 10 were taking aspirin, 1 was on dual therapy with aspirin/apixaban, and 2 were not on any antithrombotic treatment at the time of the event. Notably, 1 patient with a platelet count of 1388 × 10^9^/L and a VWF:RCo of 0.27 IU/mL underwent a bone marrow biopsy without any bleeding. Another patient with extreme thrombocytosis (1064 × 10^9^/L), but normal VWF profile, had an uneventful cesarean section.

We evaluated baseline factors associated with increased bleeding risk. Use of antiplatelet therapy showed a trend toward bleeding risk (*P* = .07) in univariate analysis. While bleeding events were more frequent in females (9/25; 36%) compared with males (4/14; 28%), this difference was not statistically significant.

Surprisingly, a higher proportion of patients experienced at least 1 bleeding episode with a VWF:RCo/Ag ratio ≥0.7 (4/7; 57.1%) compared with those with a ratio <0.7 (9/23; 28.1%; *P* = .01). Considering the small number of patients, the significance of this is unclear. The majority of patients in both groups were on antithrombotic treatment at the time of bleeding event. Three out of 4 patients who experienced bleeding events with a VWF:RCo/Ag ratio ≥0.7 were on aspirin, whereas of 9 patients with ratio <0.7 who experienced bleeding events, 7 were on aspirin, one patient was on aspirin and apixaban, and one did not receive any antithrombotic treatment. No other baseline clinical or laboratory characteristics were significantly associated with increased bleeding risk in either univariate or multivariate analysis, including platelet count, VWF:RCo, and VWF:Ag.

**Figure 1:**
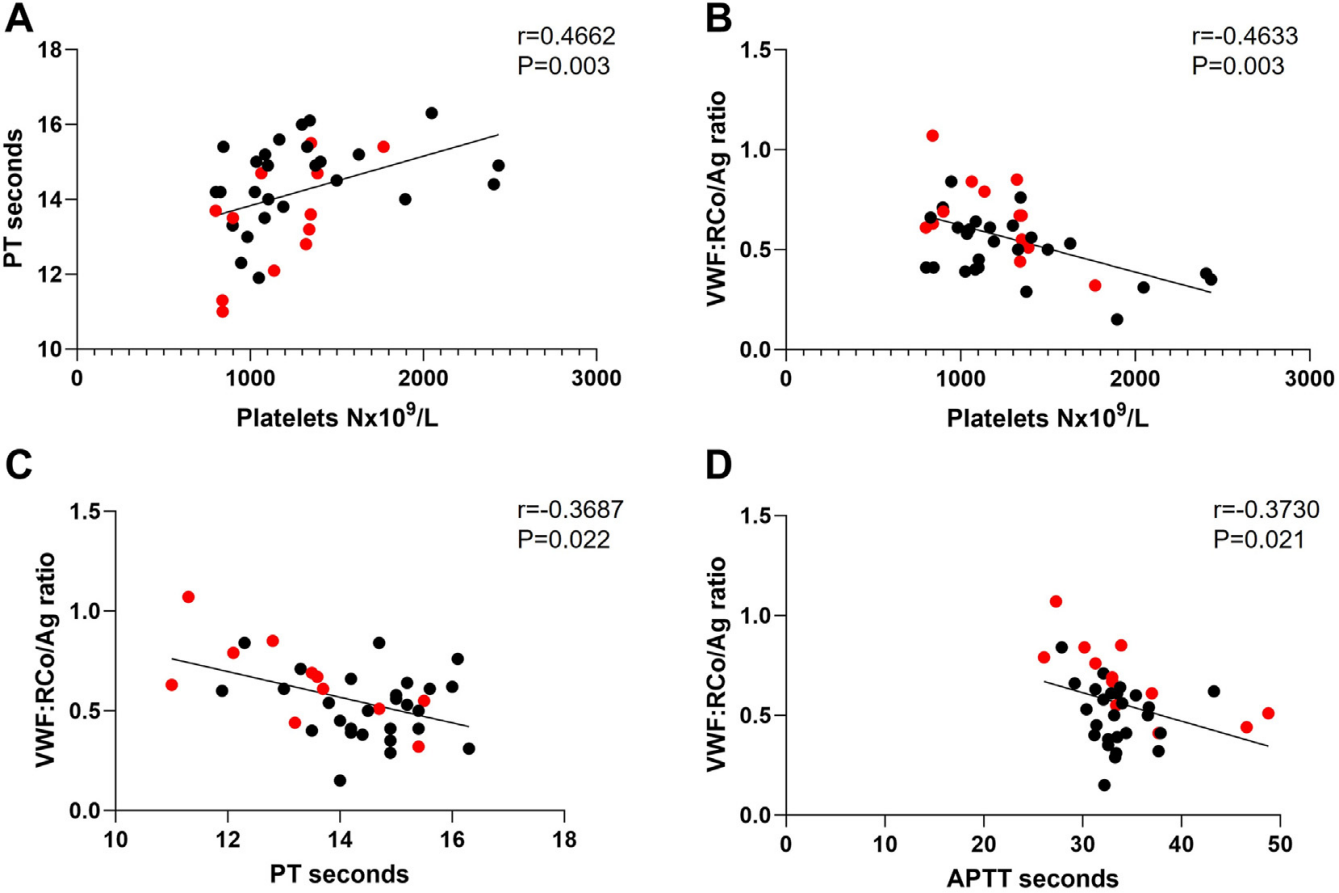
Prothrombin time (PT) shows positive correlation with platelet count (A), while the von Willebrand factor ristocetin cofactor/antigen (VWF:RCo/Ag) ratio shows an inverse correlation with platelet count (B), PT (C), and activated partial thromboplastin time (aPTT; D) in patients with myeloproliferative neoplasm and thrombocytosis at baseline. Red dots in each graph indicate patients with bleeding events. Analysis was performed with Spearman’s correlation.

**Figure 2:**
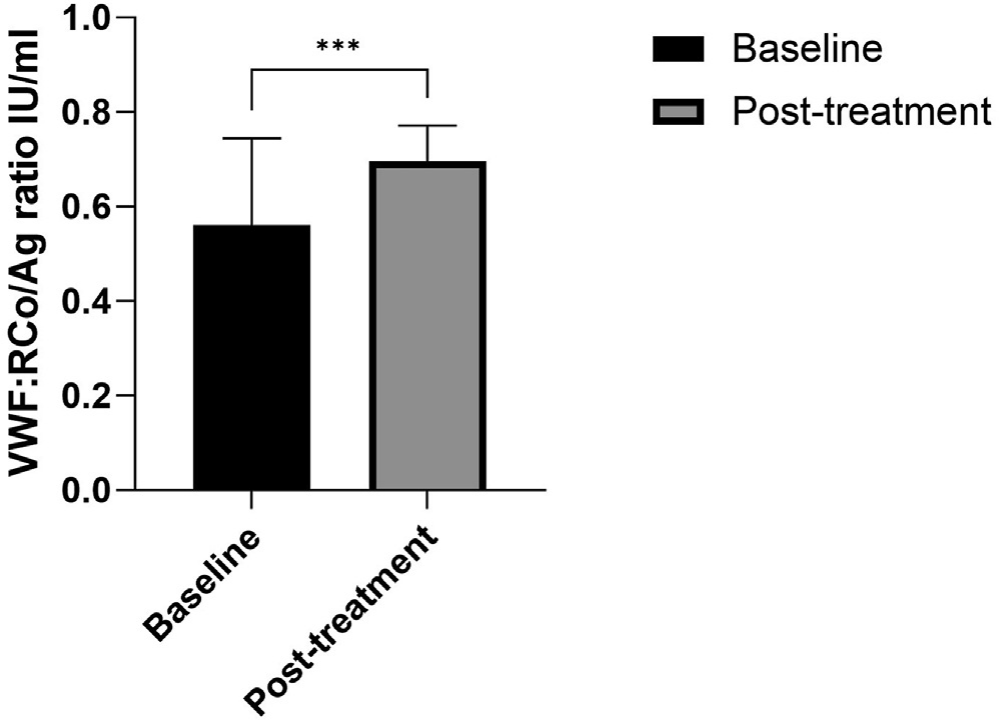
Treatment with either cytoreduction or venesection significantly increases the von Willebrand factor ristocetin cofactor/antigen (VWF:RCo/Ag) ratio. Analysis was performed with the Wilcoxon matched-pairs signed-rank test. ∗∗∗ *P* = .001 according to the Mann–Whitney U-test.

We identified significant inverse correlations between baseline VWF:RCo/Ag ratio and platelet count (*P* = .003; *r* = −.46), baseline aPTT (*P* = .02; *r* = −.37), and baseline PT (*P* = .009; *r* = −.447). Additionally, baseline platelet count and PT were significantly correlated (*P* = .003; *r* = .466; Figure 1). Baseline PT remained significantly correlated with VWF:RCo/Ag ratio post treatment of cytoreduction or venesection (*P* = .02). Post treatment, mean VWF:RCo/Ag ratio significantly increased from 0.55 at baseline to 0.71 IU/mL (*P* = .0009; Figure 2), and there was a significant decrease in platelets count, white blood cell (*P* ≤ .0001), and hemoglobin (*P* = .03). In multivariate analysis, there was a trend toward a significant association of VWF:RCo/Ag ratio < 0.7 with PT, but it did not reach significance (*P* = .08), probably due to small sample size.

Extreme thrombocytosis in MPNs presents considerable management challenges due to the complex interplay between thrombotic and bleeding risks. Our study of 39 patients with various MPN diagnoses highlights the clinical heterogeneity observed in this condition, emphasizing the need for individualized risk assessment and management strategies. Despite extreme platelet counts (median, 1168 × 10^9^/L; range, 800-2435 × 10^9^/L), bleeding outcomes varied widely. Our findings suggest that platelet count alone is not a reliable predictor of bleeding risk. This finding is consistent with previous studies [12,16].

AVWS is characterized by a qualitative defect in VWF, akin to some forms of type 2 VWD, which can occur in MPN due to: (1) High platelet counts causing absorption and sequestration of high-molecular-weight VWF multimers [8,[30], [31], [32]]. (2) Enhanced proteolytic cleavage of VWF by *ADAMTS-13* [[33], [34], [35]].

A retrospective cohort study of 565 ET patients identified that platelet counts > 1000 × 10^9^/L, splenomegaly, and leukocyte counts > 11 × 10^9^/L were significantly associated with bleeding risk [11]. Conversely, a subsequent larger analysis of ET and PFMF patients reported that only previous hemorrhage and aspirin use were independent risk factors for bleeding, while thrombocytosis alone did not predict bleeding risk [12].

Interestingly, a study from the Mayo Clinic found an association between acquired FV deficiency and MPNs, which may influence bleeding risk in these patients [36]. In that study, the cause of acquired FV deficiency remained uncertain. The authors suggested FV adsorption by the increased megakaryocytic-myeloid mass, impaired hepatic synthesis due to liver hematopoiesis, and FV inhibitors. However, they did not identify a specific FV inhibitor, and no significant correlation was found between FV activity and MPN features, nor with liver function markers. One of the patients in our cohort, with significant bleeding manifestations, including a postexertional quadriceps hematoma, was found to have low FV and dysfibrinogenemia. The significant correlations found between PT and the VWF:RCo/Ag ratio and platelet count are unexplained and of unknown significance.

Aspirin and other antiplatelet agents affect VWF function and bleeding tendency, potentially confounding any association between VWF:RCo/Ag ratio and bleeding outcomes [37]. A recent retrospective analysis of 829 patients with *BCR-ABL*–negative MPN showed that antiplatelets and anticoagulation significantly increase the risk of bleeding without affecting mortality [30]. In our cohort, almost all patients with hemorrhagic symptoms were on aspirin at the time of the bleeding event. This highlights the importance of carefully selecting patients for whom the benefits of antithrombotic treatment outweigh the associated bleeding risks.

The main limitations of this study include its retrospective nature, reliance on documentation, some missing laboratory data, and small sample size. Additionally, a level of VWF:RCo/Ag ratio indicating the presence of clinically relevant dysfunctional VWF in AVWS has not been defined, and we have used 0.7 for this purpose. Although this ratio is derived from congenital VWD, it allows for comparison with other studies of AVWS, where 0.7 or 0.6 have been used for this purpose [9,[23], [24], [25], [26]].

In conclusion, platelet count alone is not a reliable predictor of bleeding risk in MPN patients. Antithrombotic therapy, particularly aspirin and anticoagulation, increases bleeding risk and should be prescribed with caution in patients with MPN. In our study, VWF activity and antigen ratios did not predict bleeding, although these and PT may help refine bleeding risk stratification. Given the clinical heterogeneity of MPNs, a personalized approach integrating laboratory findings, bleeding history, and thrombotic risk factors is essential for optimal management.
