# Supplementary material for: Thrombocytosis and bleeding in myeloproliferative neoplasms: exploring clinical diversity and risk of acquired von Willebrand syndrome—insights from a UK center
Source: Res Pract Thromb Haemost. 2025 Jun 24;9(5):102954. doi: 10.1016/j.rpth.2025.102954 (PMC12284493; doi:10.1016/j.rpth.2025.102954)
Supplement: Supplementary Material [file mmc1.docx]

**Supplementary figure 1. Von Willebrand factor multimers analysis shows normal pattern.**

Von Willebrand factor (VWF) multimers were assessed in 18/39 patients from the study cohort and showed normal multimer pattern. Each multimer gel was run with same reference plasma and samples from 5 patients. VWF multimers of 10/18 patients are shown in the figure.

**P6**

**P7**

**Control**

**P8**

**P9**

**P10**


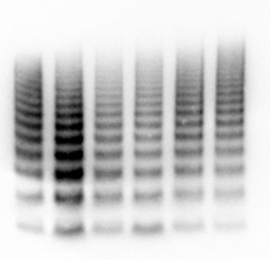

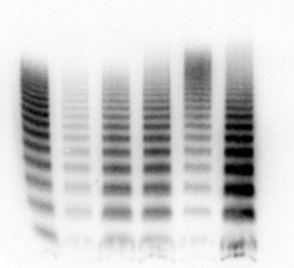


**P1**

**P2**

**P3**

**P4**

**P5**

**Control**
